# A new technique of paraffin-embedding of formalin-fixed nail sample, obtained by tangential excision - potato as guide mold^☆^

**DOI:** 10.1016/j.abd.2023.01.005

**Published:** 2023-08-30

**Authors:** Laura Bertanha, Cristina Diniz Borges Figueira de Mello, Ingrid Iara Damas, Rafael Fantelli Stelini, Nilton Di Chiacchio, Maria Letícia Cintra

**Affiliations:** a Faculdade de Ciências Médicas, Universidade Estadual de Campinas, Campinas, SP, Brazil; b Hospital do Servidor Público Municipal, São Paulo, SP, Brazil

Dear Editor,

The specimen obtained by tangential excision of the nail bed/matrix is thin and fragile and often bends after being immersed in a fixative medium. This makes it difficult to guide the orientation and paraffin embedding of the sample. The epidermal surface is normally easily identifiable on skin biopsies due to a shiny dermis. However, biopsies of the nail matrix or nail bed usually lack a nail plate, which, if present, could help identify the top of the specimen. Without the nail, both tissue surfaces are shiny.^1^ Consequently, due to an embedding defect, the pathologist has trouble identifying the different regions of the nail unit and analyzing the morphological changes.^2^

Techniques for affixing the material to the paper on which the nail apparatus was drawn have been described. They aim to guide the epithelial portion and the site from which the biopsy was obtained.^2^, ^3^, ^4^ However, even if all these techniques produce a clear distinction of the epithelial portion and adequate signaling of the biopsy site, loss of orientation may occur at the time of tissue paraffin embedding. If the orientation is compromised, for example, the normal epidermis can simulate a papilloma, resulting in misdiagnosis.

We aimed to evaluate the effectiveness of specimen embedding methodology, using a potato (*Solanum tuberosum*) as a guide mold, after approval of the ethics committee (CAAE: 48711821.0.3001.5442).

Two samples obtained for the investigation of nail melanonychia were selected. They were laid flat on a piece of filter paper, in the place corresponding to the drawn diagram of the nail apparatus, with the matrix epithelium facing upwards. The paper was folded to wrap the material, then stapled, and the envelope was immersed in 10% formalin.^3^ At the laboratory, the samples were removed from the filter paper, and inserted into a groove produced in a slice of potato (Fig. 1). This had previously been prepared by cutting into pieces measuring approximately 2 × 1.5 × 0.5 cm and kept immersed in 10% formalin for at least 24 hours, and up to 30 days, to avoid its softening. The piece of potato, with the tissue inside, was placed in a cassette for histological processing. Implementing this method resulted in perfect inclusion, with good longitudinal exposure of the nail components involved by the tuber cells (Figure 1, Figure 2).

**Figure 1:**
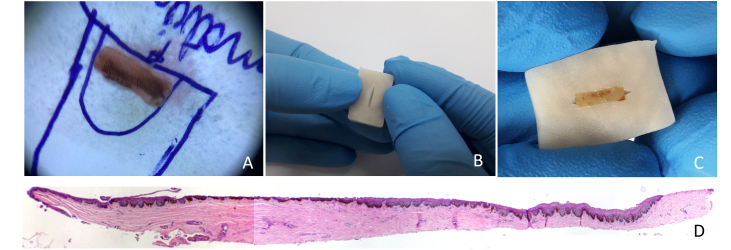
(A) The nail matrix biopsy sample is laid flat on a piece of filter paper, in the place corresponding to the drawn diagram. (B) A potato piece of 2 × 1.5 × 0.5 cm with a slit made in it by means of a scalpel. (C) The piece of potato, with the tissue inside. (D) Longitudinal section demonstrating the components of the nail apparatus. Hematoxylin & eosin, original magnification ×40.

**Figure 2:**
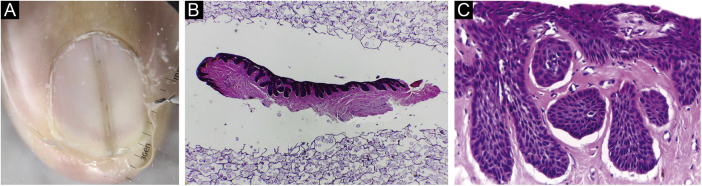
(A) Melanonychia – contact dermoscopy. (B‒C) Pigmented onychomatricoma: The specimen adequately embedded in longitudinal section. Hematoxylin & eosin, original magnification ×40, ×200.

Proper microscopic analysis of thin nail bed samples depends on suitable surgical technique, preparation, tissue fixation, and histotechnical processing, especially avoiding embedding artifacts. Inadequate surgical dissection and/or sampling or iatrogenically induced artifacts might invalidate the microscopic interpretation.^5^

In our experience, the use of potatoes as support minimizes embedding artifacts and can be a useful tool in the arsenal of care for thin and delicate tissue samples, such as those obtained from nail tangential surgeries.

## Financial support

None declare.

## Authors' contributions

Laura Bertanha: Participated in generating and analyzing the data; wrote the majority of the original draft of the paper, reviewed the pertinent raw data on which the results and conclusions of this study are based, and approved the final version of this paper.

Cristina Diniz Borges Figueira de Mello: Participated in writing the paper, reviewed the pertinent raw data on which the results and conclusions of this study are based, and approved the final version of this paper.

Ingrid Iara Damas: Participated in generating data, writing the paper and approving the final version of this paper.

Rafael Fantelli Stelini: Participated in generating data, writing the paper, reviewing the pertinent raw data on which the results and conclusions of this study are based and approving the final version of this paper.

Nilton Di Chiacchio: Participated in generating data, writing the paper, reviewing the pertinent raw data on which the results and conclusions of this study are based and approved the final version of this paper.

Maria Letícia Cintra: Participated in designing, generating, and analyzing the data; Wrote the paper and reviewed the pertinent raw data on which the results and conclusions of this study are based and approved the final version of this paper

## Conflicts of interest

None declared.
